# A retrospective observational study of the impact of 16s and 18s ribosomal RNA PCR on antimicrobial treatment over seven years: A tertiary hospital experience

**DOI:** 10.1371/journal.pone.0258552

**Published:** 2021-10-12

**Authors:** TeeKeat Teoh, Rachel McNamara, James Powell, Nuala H. O’Connell, Colum P. Dunne

**Affiliations:** 1 Department of Clinical Microbiology, University Limerick Hospital Group, Limerick, Ireland; 2 Centre for Interventions in Infection, Inflammation & Immunity (4i) and School of Medicine, University of Limerick, Limerick, Ireland; 3 Department of Medicine and Infectious Diseases, University Hospital Limerick, Limerick, Ireland; SRUC: Scotland’s Rural College, UNITED KINGDOM

## Abstract

**Background:**

Although culture-based methods remain a staple element of microbiology analysis, advanced molecular methods increasingly supplement the testing repertoire. Since the advent of 16s and 18s ribosomal RNA PCR in the 2000s, there has been interest in its utility for pathogen detection. Nonetheless, studies assessing the impact on antimicrobial prescribing are limited. We report a single-centre experience of the influence of 16s and 18s PCR testing on antimicrobial treatment, including a cost-analysis.

**Methods:**

Data were collected retrospectively for all samples sent for 16s and 18s PCR testing between January 2014 and December 2020. Results were compared to any culture-based result. Assessment focused on any change of antimicrobial treatment based on PCR result, or use of the result as supportive evidence for microbiological diagnosis.

**Results:**

310 samples relevant to 268 patients were referred for 16s/18s rRNA PCR testing during the period. Culture was performed for 234 samples. Enrichment culture was performed for 83 samples. 82 of 300 samples sent for 16s PCR had positive results (20.8%). When culture was performed, enrichment reduced the outcome of 16s PCR only positive results (4/36 [11.1%] versus 14/35 [40.0%], p = 0.030 where a pathogen found). 18s PCR yielded 9 positive results from 67 samples. The 16s PCR result influenced antimicrobial change for 6 patients (2.2%). We estimated the cost for 16s PCR testing to result in one significant change in antimicrobial therapy to be €3,340. 18s PCR did not alter antimicrobial treatment.

**Conclusion:**

There was limited impact of 16s PCR results on antimicrobial treatments. Relevance to practice was affected by relatively long turn-around-time for results. Utility may be increased in specialised surgical centres, or by reducing turn-around-time. Enrichment culture should be considered on samples where 16s PCR is requested. There remains limited evidence for use of 18s PCR in clinical management, and further studies in this area are likely warranted.

## Background

Molecular approaches play an increasingly important role in infectious disease diagnostics [1]. Although culture-based methods remain a constant element of microbiology analysis, advanced molecular methods are steadily supplementing the testing repertoire [2, 3]. Molecular methods have both the potential for rapid identification of potential pathogens and detection of fastidious or slow growing organisms [4]. Advances in molecular diagnostic technologies have prompted evolution of syndromic testing, where simultaneous testing of pathogens can be performed for specific clinical syndromes. Syndromic panels have replaced standard culture methods in some circumstances; for example, in diagnostic use for sexually transmitted illness and enteric pathogens [3, 5]. Although, some limitations exist for these panels, the rapid turn-around-time assists in clinical diagnosis of severe infection with actionable case management; for example, in the detection of central nervous system pathogens with the FilmArray ME panel (Biomerieux, Marcy-l’Etoile, France) [6].

Clinical management and antimicrobial therapy are generally tailored to pathogens detected via culture. However, culture-negative samples are often encountered in the clinical microbiology laboratory, and this impacts treatment options and outcomes of infectious diseases negatively. For example, in prosthetic joint infections Tan et al. observed 1-year treatment success was only 69.2% in cases where the causative aetiology was unknown [7]. Furthermore, Siciliano et al. reported mortality for community-acquired culture-negative endocarditis as being not higher than culture positive endocarditis but significant at 33.0% [8]. Tenforde et al., in a study of meningitis in Botswana—a region with high endemicity of HIV, reported that mortality for culture-negative meningitis was comparable to pneumococcal and tuberculous meningitis. They concluded that this was due somewhat to the limited diagnostic services available in routine clinical care [9]. These studies emphasise the importance of laboratory confirmation of pathogens in serious infections.

Since its advent in the 2000s, there has been interest in 16s and 18s ribosomal RNA (rRNA) PCR for detection of bacterial and fungal pathogens, resulting in use as pan-bacterial and pan-fungal molecular diagnostic tests, respectively. Of particular interest is the diagnosis of potential pathogens in culture-negative or apparently sterile samples.

Previous studies have shown that 16s PCR has relatively good sensitivity and specificity [10–14]. Although 18s PCR studies are more limited, Wagner et al. reported reasonable concordance between 18s PCR and culture-based methods [15]. Nonetheless, studies assessing the role of 16s and 18s PCR results with regard to influence on antimicrobial prescribing are more limited. Moreover, 16s and 18s PCR are not performed routinely in many clinical laboratories and for many centres remain a specialised process requiring external referral. As such, it is important to ascertain the clinical impact and value of employing 16s and 18s PCR with regard to antimicrobial stewardship and the associated costs.

Previously, we described the role of molecular technologies in the diagnosis and management of infectious diseases with direct application to individual case management [16–18]. In that context, we continue here with an analogous exploration of the effect of 16s/18s PCR on infectious disease diagnostics and subsequent impact on the clinical management of suspected infections. Additionally, we performed a cost-analysis on use of 16s and 18s PCR in our centre.

## Methods

### Setting and inclusion criteria

This retrospective study was conducted in University Hospital Limerick (UHL), a 455-bed tertiary referral centre in the Mid-West of Ireland that is part of the University Limerick Hospital group (ULHG), serving a population of circa. 473,000. All microbiological testing was performed in the ULHG Clinical Microbiology laboratory located in UHL. Data relevant to all patients who had samples sent for 16s/18s broad-range rRNA PCR between January 2014 and December 2020 were included in this study.

### Ethical approval

This study was approved by the Research Ethics Committee of University Limerick Hospital Group, Limerick, Ireland. All data accessed were anonymised and individual patient consent deemed not required.

### Laboratory processing

The ULHG Clinical Microbiology laboratory processes an average of 50,000 specimens in a year. Laboratory standard operating procedures are adapted from the UK Standards for Microbiological Investigation (UK SMI). Bacterial and fungal identification was performed primarily with matrix laser desorption/ionisation-time of flight (MALDI-TOF) (Bruker, MA, USA). Antimicrobial susceptibility testing was completed and reported based on European Committee of Antimicrobial Susceptibility Testing (EUCAST) methods and breakpoints. All culture methods are accredited to ISO15189 standards by the Irish National Accreditation Board. Enrichment culture using a cooked meat broth (Fannin, Galway, Ireland) was performed on samples labelled pus, aspirates, bone, and tissue routinely as per laboratory standard operating procedures. Samples received in blood culture bottles (e.g., pleural or ascitic fluid) were enriched using Bactec^TM^ Fos^TM^ culture supplement (Beckson-Dickson and Company, NJ, USA) and incubated in the BacT/Alert 3D system (Biomerieux, Marcy-l’Etoile, France). All enrichment cultures were incubated for 5 days.

Our centre refers all 16s/18s rRNA PCR requests to an accredited external laboratory, Micropathology Limited (Coventry, England). PCR is performed by a manual extraction process followed by a quality assured laboratory developed PCR assay. Results are compared to known sequences in GenBank^®^. Samples were referred for 16s or 18s PCR testing at the request of a Medical Microbiologist or Infectious Disease Physician, with a decision made based on the clinical scenario and diagnostic question posed. Samples were also sent for 16s and 18s PCR at the discretion of the Medical Microbiologist where sample volume was deemed inadequate for culture methods.

### Data collection

Data for samples sent for 16s and 18s rRNA PCR and culture results were collected retrospectively via the laboratory information management system (DXC/iLAB). Clinical data pertinent to the study were obtained via laboratory electronic notes, those compiled by the Medical Microbiology team, Emergency Department notes, radiological reports, and paper-based medical records. All patient data were anonymised in compliance with the General Data Protection Regulation (GDPR). Electronic and paper-based records were reviewed to assess whether a significant change in antimicrobial therapy occurred based on 16s or 18s PCR result. The cost of transport and processing of 16s and 18s PCR testing was derived from billing charges from the approved courier and reference laboratory.

We defined a significant change in antimicrobial therapy as a change to the spectrum of antimicrobials or specific antimicrobials to target specific pathogens, or a change in duration of antimicrobials, based on the 16s or 18s PCR result only. We further assessed the relevance of 16s or 18s PCR result in providing supportive microbiological diagnosis, whereby a PCR result is concordant with culture-based identification.

### Statistics

Analyses were performed using SPSS v26.0 (IBM) and p-values were calculated to ascertain statistical significance. A p-value of ≤0.05 was considered statistically significant. Chi-squared or Kruskal-Wallis tests were used for categorical data.

## Results

Between 2014 and 2020, a total of 310 samples from 268 patients were referred for 16s/18s rRNA PCR testing. 243 samples were referred for 16s PCR only, 10 for 18s PCR only and 57 for both 16s and 18s PCR. Table 1 details the breakdown of sample type. Culture was performed for 234 samples and the remaining 76 samples underwent PCR testing only. Enrichment culture was performed for 83 samples (35% of cultured samples). The mean turn-around-time from sample collection to receiving the 16s or 18s PCR result was 6.68 days (interquartile range (IQR), 3–8). Table 2 details the breakdown of positive 16s and 18s PCR results.

**Table 1:** 16s/18s PCR test requests and positivity rate per sample type.

Sample Type	16s PCR Test Requests, n	Positive 16s PCR result, n (%)	18s PCR Test Requests, n	Positive 18s PCR result, n (%)
**Musculoskeletal**	** **			
• Soft tissue fluid/abscess	23	7 (30.4)	2	0 (0)
• Skin/soft tissue biopsy	17	6 (35.3)	7	1 (14.2)
• Lymph node tissue	5	0 (0)	2	0 (0)
• Joint aspirates/fluids	46	7 (15.2)	3	0 (0)
• Joint tissue	26	3 (11.5)	3	0 (0)
• Spinal tissue	5	0 (0)	1	0 (0)
• Bone	17	1 (5.9)	5	0 (0)
**Gastrointestinal**	** **			
• Abdominal fluid/pus	12	9 (75)	6	2 (33.3)
• Abdominal tissue	2	1 (50)	2	1 (50)
• Liver aspirates	8	4 (50)	1	0 (0)
• Ascitic fluid	6	0 (0)	0	0 (0)
**Eye**				
• Conjunctival swab	2	0 (0)	1	0 (0)
• Corneal scrapping	0	0 (0)	2	1 (50)
• Vitreous fluid	4	1 (25)	1	0 (0)
**Respiratory**	** **			
• Pleural fluid	46	8 (17.4)	1	0 (0)
• Transbronchial biopsy	38	13 (34.2)	14	1 (7.1)
• Bronchoalveolar lavage	5	1 (20)	5	3 (60)
**Cerebrospinal fluid**	17	0 (0)	6	0 (0)
**Pericardial fluid**	4	0 (0)	0	0 (0)
**Whole blood**	15	0 (0)	5	0 (0)
**Others**	2	1 (50)	0	0 (0)

**Table 2:** Comparison of microbiological diagnosis by culture versus 16s/18s rRNA PCR.

**Bacterial target**	**PCR (n = 300)**	**Culture (n = 215)**
**Gram positive bacteria**		
*Staphylococcus aureus*	6	3
Coagulase negative staphylococci	3	16
*Staphylococcus sp*. (subspecies unidentified)	3	n/a
*Streptococcus pneumoniae*	2	0
*Streptococcus pyogenes*	2	1
*Streptococcus agalactiae*	1	0
*Streptococcus milleri* group	1	2
Viridian group streptococci	1	1
*Streptococcus* species	1	0
*Enterococcus sp*	5	2
*Propionibacterium sp*.	1	0
*Corynebacterium sp*.	1	0
*Bacillus sp*.	0	0
**Gram negative bacteria**		
*Klebsiella sp*	2	0
*E*. *coli*	2	1
*Serratia sp*.	1	0
*Pantoea sp*.	4	0
*Neisseria meningitidis*	1	0
*Moraxella sp*.	1	2
*Pseudomonas sp*.	3	2
*Pasteurella multocida*	1	0
*Sternotrophomonas maltophilia*	2	1
*Pantoea sp*	4	0
*Acinetobacter sp*	1	0
*Mycoplasma hominis*	1	1
**Anaerobes/Others**		
*Fusobacterium sp*.	5	0
*Bacteroides sp*.	0	1
*Clostridium sp*.	0	1
Anaerobes (not identified)	0	1
*Mycobacterium chelonae*	1	0
Mixed (bacterial target/culture)^1^^,^^2^	8	18
Total positive	62	53
**Fungal target**	**18s PCR (n = 67)**	**Culture (n = 35)**
*Candida albicans*	2	1
Other *Candida sp*	4	4
*Rhodoturula sp*.	1	0
*Aspergillus sp*.	1	1
*Penicillium sp*.	1	1

^1^ Mixed bacterial 16s PCR is defined as any report with mixed sequence results or when more than one bacterial target detected.

^2^ Mixed bacterial culture is defined as any culture report with >1 species grew.

**Fig 1:**
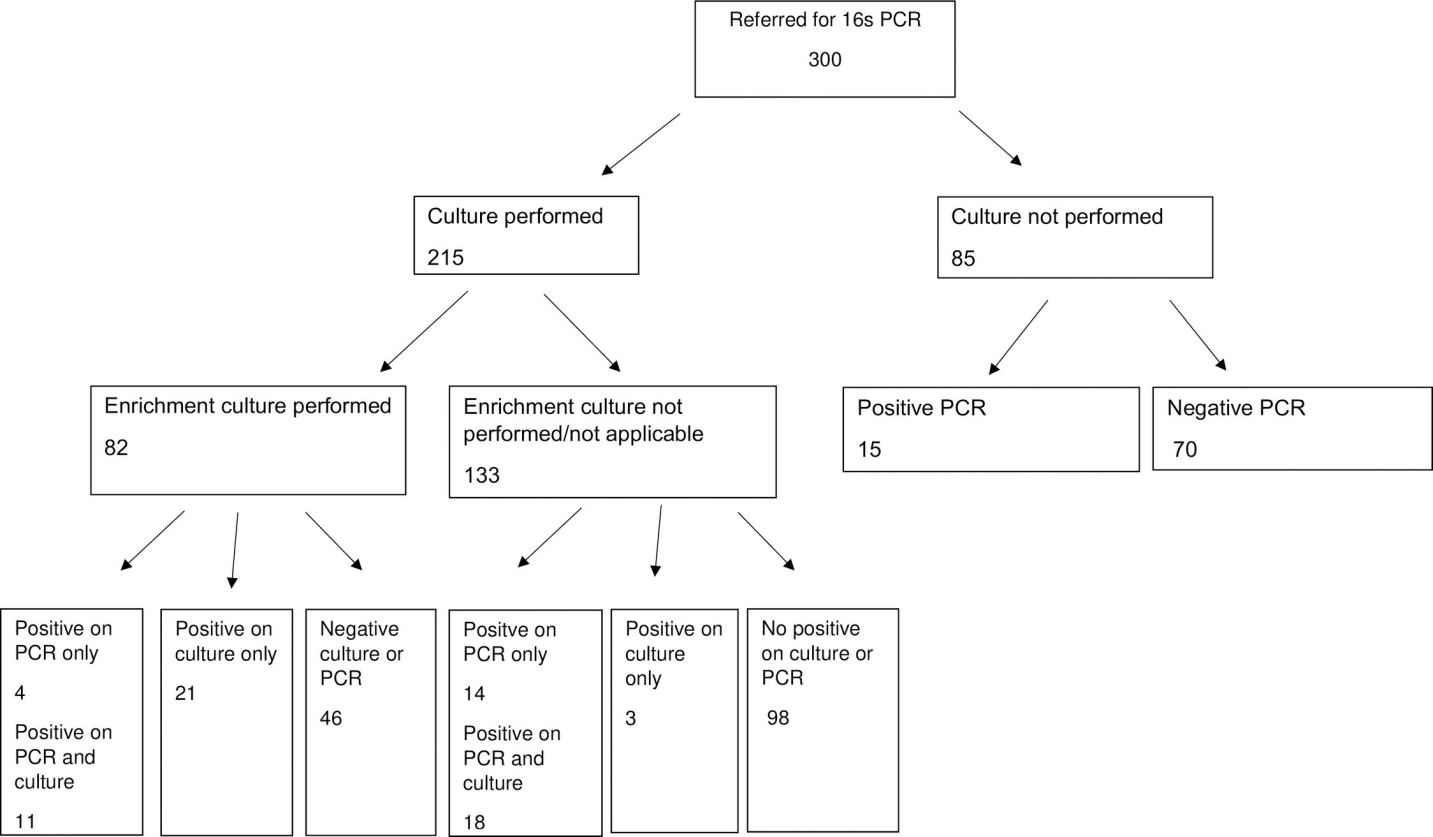
Results for samples referred for 16s PCR versus conventional culture and enrichment culture.

For samples sent for 16s PCR, culture was performed on 215 samples and not performed for 85 samples. Enrichment was completed for 82 samples. Of the 300 samples, 62 samples yielded positive targets (20.7%) using 16s PCR. Of the samples that underwent culture, 18 were 16s PCR positive only, whereas 29 samples were both culture and PCR positive. Twenty-four samples detected pathogens on culture only, of which 21 samples had undergone enrichment. Fig 1 summarises the results for samples sent for 16s PCR. Notably, 16s RNA only positive outcomes were observed less frequently in cases where samples had been subject to enrichment (11.1% enriched versus 40% unenriched, p = 0.030). We also observed that intra-abdominal samples had higher rates of positive results for 16s PCR compared to respiratory and musculoskeletal samples (45.2% versus 24.7% and 17.3%, p = 0.0036).

**Fig 2:**
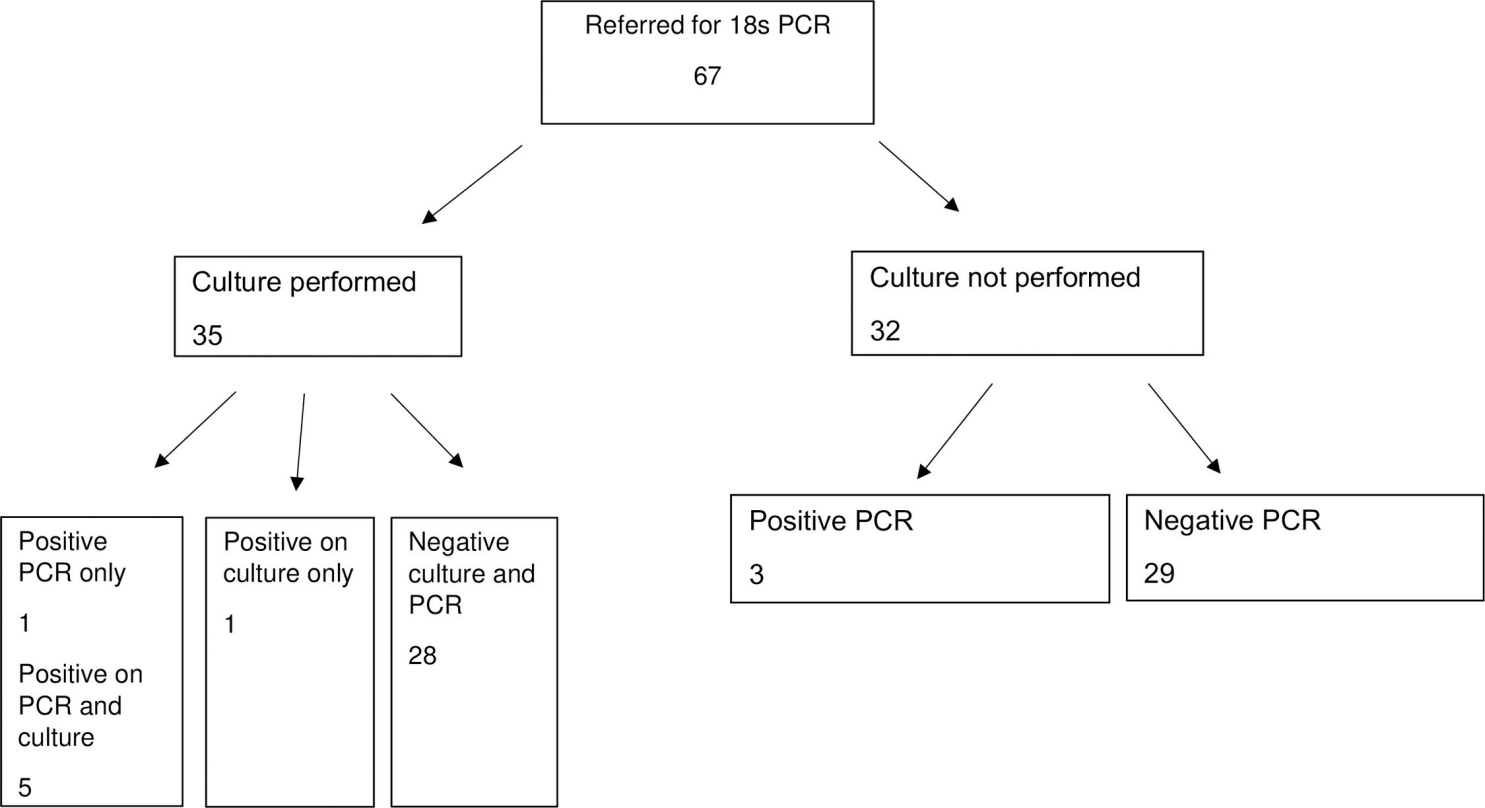
Results for samples referred for 18s PCR versus culture.

For 18s PCR, 35 out of 67 samples were cultured. Nine samples yielded positive results for possible fungal pathogens. A PCR only positive result was detected in 1 sample, 1 sample was culture-positive only, and 5 samples had similar pathogens using both culture and PCR (Fig 2).

With regard to the clinical impact of PCR testing, six patients (2.2%) experienced a significant change in their antimicrobial treatment based on 16s PCR result. We observed that 18s PCR results did not influence antimicrobial treatment at all. Similarly, negative results did not lead to observed cessation of antimicrobial treatment. On further analysis, 6 PCR positive samples provided support for microbiological diagnosis of the causative pathogen in deep seated infections.

### Cost analysis

The total cost for testing 310 samples for 16s and 18s PCR was €23,288.50. Direct cost of a single 16s or 18s PCR test is €45. Cost of delivery of samples to the reference laboratory is €21.85 per sample. In our study, the number needed-to-test for 16s PCR to result in a significant antimicrobial change is 50, translating to an expenditure of €3340. For 18s PCR, the cost of testing (including transportation costs) was €4,478.95, but did not lead to any significant change in antimicrobial use.

## Discussion

In our study, we observed limited utility for use of 16s and 18s PCR testing as an adjunct to culture methods for infectious disease diagnostics. Benefit was limited to 16s PCR detection of fastidious organisms that are difficult to culture; illustrated by the detection of *Neisseria meningitidis* and *Fusobacterium* species via 16s PCR molecular methods alone. We also observed some, but limited, 16s PCR support for microbiological diagnosis in 2 cases of *Streptococcus pneumoniae* empyema.

The impact on antimicrobial treatment was minimal. We observed only 6 (2.2%) cases where 16s PCR resulted in a significant change of antimicrobial treatment. Our findings are contrary to those from another Irish centre where in 2018 they reported successful de-escalation of therapy in 21% of patients upon adoption of 16s PCR diagnostics in their centre [19]. In 2021, Ursenbach et al. also reported successful change in therapy for 32% of cases with a positive 16s target in a single French centre [20]. However, these hospitals provided quaternary clinical services with specialist neurosurgical and cardiothoracic departments, in contrast to our general hospital model.

One important contributor to our findings was the long turn-around time from sample collection to reporting, with a mean time of 6.8 days. This is significantly longer than reported by Ursenbach et al., who benefited from 16s PCR technology availability within onsite diagnostic services [20]. Previously, we reported the use of molecular diagnostics with rapid turn-around-times that led to actionable case management; positive use of Abbott ID NOW™ in our Emergency Department leading to reduced admissions and reduction in healthcare associated influenza [18]. Thus, it is reasonable to argue that long turn-around time from sample collection to reporting time (and further delays to clinician feedback of result) adversely affects test utility in a majority of cases. The contrasting results between our study and those of both Ursenbach et al. and O’Donnell et al. illustrate the conundrum with 16s or 18s PCR testing as, although clinical influence is occasionally observed, the lengthy turn-around time for these specialised tests negates relevance in clinical management. Aggarwal et al. argued a similar point for more rapid turn-around-time of 16s PCR to improve clinical impact [21]. We similarly failed to observe any useful impact on clinical management in cases of negative 16s and 18s PCR results.

One further confounding factor for the impact of 16s and 18s PCR on clinical care is the importance of a robust feedback loop of results to primary clinicians. Timbrook et al. report in their meta-analyses that the benefit of rapid molecular diagnostics on mortality in blood stream infection only occurs in the setting of an antimicrobial stewardship (AMS) programme [22]. O’Donnell et al. alludes to this fact as, in their centre, the Clinical Microbiology service provides ward-based consults and plays a leadership role in their AMS programme [19]. Due to a delayed turn-around-time and the complexity of interpretation of microbiology results, a robust reporting system encompassed into an AMS programme is likely key to using 16s and 18s PCR to impact clinical care. In our centre, laboratory paper reports were relied on to feedback results unless the result was highlighted to the Medical Microbiologist as warranting a phone call to the primary clinician. This may explain some differences in our study compared to the studies by O’Donnell et al and Ursenbach et al.

Notably, Ursenbach et al. reported significant changes in antimicrobial treatment choice following positive 16s PCR for CSF samples, albeit in only 21 cases [20]. In our centre, prior to the introduction of the FilmArray ME Panel (bioMérieux, Marcy l’Étoile, France) in October 2017, bacterial testing for CSF was limited to culture and agglutination test methodologies. In our study, we found use of 16s PCR for CSF samples failed to yield any positive results, versus 11% in the Ursenbach *et al*. study. Since the introduction of the FilmArray ME panel in our laboratory routine testing repertoire, we have observed its utility in guiding clinical management and cessation of antibiotics [16]. This augments our point that rapid turn-around-time of reporting of results plays an important role in impacting clinical management. We conclude that 16s PCR testing in CSF may have a more limited or specialised role considering the advent of syndromic testing PCR panels.

Furthermore, in our data, performance of enrichment improved yield of conventional microbiology results to such a degree that 16s PCR outcomes were largely redundant. The advantage of enrichment culture is that many laboratories are able perform it locally, reducing need for external laboratories or development of comparatively expensive in-house PCR capabilities.

Lastly, we failed to note any clinically significant results obtained in samples that were sent for PCR testing only, with no culture performed. In our study, 27.4% of samples were referred for PCR testing due to clinician suspicion of multiple pathogen involvement (e.g., fungal, bacterial or mycobacterial) or for samples with minimal volume or small sample size. However, although a positivity rate for this was 17.6%, it did not lead to any meaningful change in clinical management. Also, there were no meaningful results for 18s PCR that impacted clinical management. Nonetheless, we observed a high specificity of results. In conclusion, there remains sparse evidence of the impact of 18s PCR testing in clinical management, and further studies in this area are likely warranted.

The cost of 16s and 18s PCR testing in our centre, although relatively low for a 7-year period, is not insignificant. Importantly, in our hands, an expenditure of €3340 on 16s PCR was required to result in a single significant change in antimicrobial therapy. Aggarwal et al. similarly observed the cost for a 16S PCR positive/culture-negative result to impact an antimicrobial prescription equated to £4041.76 [21]. To our knowledge, no studies have assessed the cost of culture-negative infective syndromes specifically and, so, the cost-benefit of 16s and 18s PCR use remains undetermined.

The limitations of our study relate to its retrospective and single centre design, and the observed lack of relevance of 16s and 18s PCR results in clinical management may be due to other multi-factorial reasons beyond the scope of this study. Due to the lack of electronic prescribing records, we did not assess individual antimicrobial duration in this study. Indeed, an element of selection bias cannot be ruled out completely due to the deficiency of information regarding the decision to request 16s and 18s PCR tests on specimens There may be subgroups of sample types amenable to 16s and 18s PCR, but our study was not designed to study this. However, the outcome of this study remains widely generalisable, and may be a useful resource for clinical microbiologists and infectious disease specialists interested in reviewing, or enhancing, existing molecular diagnostic services with regards to clinical impact.

In conclusion, 16s PCR remains an interesting adjunct diagnostic test in the diagnosis of infectious diseases, albeit with high associated cost as evidenced by the number of tests needed to impact antimicrobial treatment. It is likely that its routine use may be more beneficial to specialised surgical centres rather than to general patient populations, and relatively long turn-around-time affect its clinical relevance negatively. Reducing the turn-around-time may increase its clinical usefulness but may require investment to develop local testing capacity. There remains limited evidence of the role of 18s PCR in clinical management and further studies in the area are likely warranted. Commercial syndromic testing panels with rapid results have supplanted some of the utility of 16s and 18s PCR testing, and the rapid advancement of next generation sequencing may further reduce its future relevance.
